# Prediction of the binding affinities of peptides to class II MHC using a regularized thermodynamic model

**DOI:** 10.1186/1471-2105-11-41

**Published:** 2010-01-20

**Authors:** Andrew J Bordner, Hans D Mittelmann

**Affiliations:** 1 Mayo Clinic, 13400 East Shea Boulevard, Scottsdale, AZ 85259, USA; 2 School of Mathematical and Statistical Sciences, Arizona State University, P.O. Box 871804, Tempe, AZ 85287, USA

## Abstract

**Background:**

The binding of peptide fragments of extracellular peptides to class II MHC is a crucial event in the adaptive immune response. Each MHC allotype generally binds a distinct subset of peptides and the enormous number of possible peptide epitopes prevents their complete experimental characterization. Computational methods can utilize the limited experimental data to predict the binding affinities of peptides to class II MHC.

**Results:**

We have developed the Regularized Thermodynamic Average, or RTA, method for predicting the affinities of peptides binding to class II MHC. RTA accounts for all possible peptide binding conformations using a thermodynamic average and includes a parameter constraint for regularization to improve accuracy on novel data. RTA was shown to achieve higher accuracy, as measured by AUC, than SMM-align on the same data for all 17 MHC allotypes examined. RTA also gave the highest accuracy on all but three allotypes when compared with results from 9 different prediction methods applied to the same data. In addition, the method correctly predicted the peptide binding register of 17 out of 18 peptide-MHC complexes. Finally, we found that suboptimal peptide binding registers, which are often ignored in other prediction methods, made significant contributions of at least 50% of the total binding energy for approximately 20% of the peptides.

**Conclusions:**

The RTA method accurately predicts peptide binding affinities to class II MHC and accounts for multiple peptide binding registers while reducing overfitting through regularization. The method has potential applications in vaccine design and in understanding autoimmune disorders. A web server implementing the RTA prediction method is available at http://bordnerlab.org/RTA/.

## Background

Class II MHC is an essential protein complex in the adaptive immune system that is involved in activating helper T cells. It is found on the surfaces of specialized immune system cells, such as dendritic cells and macrophages, where it binds fragments of extracellular peptides and presents them to CD4^+ ^helper T cells. Class II MHC is highly polymorphic and each allotype generally has different peptide binding preferences. The computational prediction of such binding affinities can be applied to the important problem of finding promiscuous epitopes that bind to multiple allotypes for use in rational vaccine design. Also these predictions may aid in understanding autoimmune diseases, many of which have been linked to particular class II MHC alleles [1].

Characteristic properties of peptide binding to class II MHC molecules can be inferred from available high-resolution X-ray structures of peptide-MHC complexes. The binding cleft in class II MHC is open at both ends so that it generally binds longer peptides, 15-25 residues in length, with the peptide N- and C-terminii extending out of the cleft. This is in contrast to class I MHC, which has a peptide binding cleft that is closed at both ends so that it only accommodates shorter peptides of 8-11 residues. An examination of X-ray crystal structures of peptide bound class II MHC molecules reveals that the peptides bind in an extended polyproline II helix with the conserved backbone structure maintained by conserved hydrogen bonds with the MHC [2]. Peptide side chains within a contacting nine residue core segment of the peptide bind into pockets on the MHC surface [1]. Many of the polymorphic residues occur in these pockets and so determine the distinctive peptide binding preferences of each MHC allotype. Although the core residues of the bound peptide assume similar backbone conformations, each peptide can generally bind in multiple registers. This makes the prediction of peptide binding affinities for class II MHC particularly challenging since, unlike class I MHC, all possible binding registers must be considered.

Prediction methods for class II epitopes remains an active area of research, motivated both by their biomedical importance and the difficulty of achieving high accuracy. Early prediction methods fit the total peptide binding energy [3-6], binding motif [7], geometric average binding affinity [8], or sequence alignment profile [9] in a particular register to a linear combination of contributions from individual residues, and represented them as binding profile matrices. The scores for all possible peptide binding registers were calculated and either the maximum value or sum were used as a total peptide binding score. Later methods employed various machine learning and data fitting approaches to prediction including partial least squares (PLS) [10,11], Gibbs sampling [12], linear programming [13], Support Vector Machines (SVMs) [14-16], and kernel methods [17]. One prediction method, called SMM-align, successfully combined two methods, binding profile matrices and Gibbs sampling [18].

We introduce a new model for predicting binding affinities of peptides to class II MHC called the Regularized Thermodynamic Average, or RTA, model. Three features are incorporated into the model: (1) independent binding of individual peptide residues so that the total binding energy is a sum of the binding energies of each core peptide residue, (2) a thermodynamic average over all possible peptide binding registers, and (3) regularization of the model to prevent overfitting. Feature #1, the approximate independence of peptide binding on peptide residue interactions, is supported experimentally [3] and is a basic assumption in most prediction models to date. Indeed, it is the primary basis for binding profile methods. The importance of contributions of suboptimal peptide binding registers to the total binding affinity motivates Feature #2. Experimental studies have found such cases in which a peptide binds with significant affinity to class II MHC in multiple registers [19-21]. In fact, such peptide binding in multiple registers may be involved in the origin of autoimmune diseases [22]. The thermodynamic average in the RTA model insures that contributions from suboptimal binding registers, which may be significant, also contribute to the total binding affinity. Regularization (feature #3) is especially important in prediction models that contain a relatively large number of parameters, such as ours. Regularization is implemented in RTA using an L^1 ^penalty function, like in lasso regression [23]. This has the advantage over alternative penalty functions, such as the L^2 ^penalty employed in SMM-align, that many coefficients vanish at lower cutoff values so that the number of model parameters is reduced. In addition, unlike in many previous prediction methods, the P1 residue for peptides binding to HLA-DR is not restricted to be hydrophobic in the RTA model. Finally, we use deterministic local optimization methods to fit the model parameters, which we found to converge to better solutions than commonly employed stochastic optimization methods.

The RTA prediction method was tested using cross-validation sets from an SMM-align study [18], for comparison with results from 9 different prediction methods examined in that study, and also using larger cross-validation sets created from binding data from the IEDB database [24]. The RTA method was also evaluated using additional peptide binding data sets from Lin *et al*. 2008 [25] and El-Manzalawy *et al*. 2008 [26]. A novel method to efficiently create data sets with minimal sequence overlap was developed to create the new cross-validation sets. The performance of RTA in predicting peptide binding affinities and in predicting the correct peptide core binding residues were tested. Finally, we examined the relative contribution of suboptimal peptide binding registers to the overall binding affinity.

## Methods

### Peptide-MHC binding data set

Peptide binding data for the 16 MHC allotypes in Table 1 were downloaded from the IEDB database http://www.immuneepitope.org[24]. All IC_50 _values from either radioactively or fluorescently labeled peptide competition binding assays were collected. The median value was used for the few cases in which multiple experimental values were measured. The total numbers of data for each allotype are given in Table 1. These datasets were then divided into minimally overlapping 5-fold cross-validation sets using the method described in "Creating independent cross-validation sets".

**Table 1:** Cross-validation prediction results for the IEDB data sets

MHC allele	AUC	RMS error (kcal/mol)	Correlation coefficient	Number of data
DRB1*0101	0.749	1.43	0.530	5648
DRB1*0301	0.762	1.46	0.425	837
DRB1*0401	0.715	1.72	0.340	1014
DRB1*0404	0.792	1.38	0.487	617
DRB1*0405	0.757	1.35	0.442	642
DRB1*0701	0.790	1.62	0.484	833
DRB1*0802	0.747	1.34	0.412	557
DRB1*0901	0.711	1.68	0.369	551
DRB1*1101	0.753	1.45	0.450	812
DRB1*1302	0.765	1.64	0.464	636
DRB1*1501	0.736	1.53	0.438	879
DRB3*0101	0.825	1.13	0.425	483
DRB4*0101	0.799	1.33	0.522	664
DRB5*0101	0.732	1.57	0.434	835
H2-IA^b^	0.828	1.15	0.556	526
H2-IA^d^	0.814	1.53	0.563	306

### Prediction Model

As mentioned above, the prediction of peptide - class II MHC binding affinity involves not only estimating the affinity of the bound core portion of the peptide but also accounting for multiple binding registers. The model begins with the approximation that the contributions to the total binding free energy of binding individual peptide residues to each MHC pocket are approximately independent. This assumption has been tested experimentally [3] and is the basis of matrix-based prediction methods in which the total peptide-MHC binding energy is a sum of pocket specific residue binding energies [5]. Furthermore, X-ray structures of peptide - class II MHC complexes show that the 9-residue core bound portions of the peptides adopt similar backbone conformations so that the total score over an ungapped 9-residue segment of the peptide sequence is appropriate.

The peptide-MHC binding data from competition experiments are given as IC_50 _values. IC_50 _is not a direct measure of binding affinity but it can be related to the inhibition constant, K_i_, through the Cheng-Prusoff equation(1)

[L] is the concentration of the free labeled peptide and K_d _is its binding affinity to the MHC molecule [27]. It is assumed that the labeled peptide concentration is sufficiently low, *i.e*. [*L*] = *K*_*d*_, so that *K*_*i *_≈ IC_50_. This is an implicit assumption in other prediction methods as well. According to this approximation, experimental binding affinities were calculated as Δ*G*_exp _= - *kT *log(IC_50_), in which kT is the Boltzmann constant times the absolute temperature, which is approximately 0.586 kcal/mol at room temperature.

Each peptide can bind the MHC molecule in different registers defined by which 9-residue core peptide segment contacts the MHC. The length of peptide k is denoted by L(k). The register will be denoted by the index M, which varies from 0 for the first 9 residues as the core segment to L(k) - 9 for the last 9 residues as the core segment. The sequence of peptide k is represented by matrix elements , which are equal to 1 if residue type j appears at position i in the peptide sequence and are equal to 0 otherwise. The affinity of peptide k binding to the MHC molecule in register M is then the linear function(2)

in which the coefficients β_ij _are to be fit using the experimental data.

The apparent total binding affinity for peptide k can be calculated as(3)

in which  is the fraction of bound peptides binding in register M and  is the affinity for binding in register M, calculated in the model using Eq. 2. An entropic contribution to the free energy in Eq. 3 was omitted because it did not improve prediction performance (data not shown). The fraction of peptide bound in a particular register is related to the binding affinities in Eq. 2 by(4)

[p^(*k*)^], [MHC], and [ - MHC] are the concentrations of unbound peptide, unbound MHC and peptide bound to the MHC in register M, respectively.  is the dissociation constant for peptide k binding to the MHC in register M. By combining Eqs. 3 and 4 one arrives at an expression for the total binding affinity as an exponentially-weighted average of the affinities for the peptide binding in different registers(5)

Many prediction methods for peptide-MHC binding make the further approximation that the largest binding affinity dominates the sum in Eq. 5 so that only the largest binding affinity over all possible registers is included in the prediction model. Because suboptimal terms are comparable in magnitude to the optimal term in many cases, we will retain all terms in Eq. 5.

The final component in the prediction model is regularization. This is needed to avoid overfitting, since the model contains 180 parameters, which is relatively large compared to the number of binding data for each MHC type. Regularization is accomplished by imposing an upper bound on the L^1 ^norm of the coefficient amplitudes(6)

with the cutoff parameter t. This type of constraint is also used in lasso regression [23] and has the desirable property that as the cutoff t is lowered, an increasing number of coefficients become zero so that the number of nonzero parameters in the model is reduced.

By combining these components, the binding affinity for peptide k, Δ*G*^(*k*)^, is calculated as(7)

The parameters in this model, {*β*_ij_, *i *= 1, K, 9, *j *= 1, K, 20}, are fit to the experimental binding affinities, , by minimizing the mean square error (MSE)(8)

subject to the constraint in Eq. 6.

### Optimization method

To solve the optimization problem minimizing Eq. 8 under the constraint in Eq. 6 one first splits the variable β_ij _into the difference of two nonnegative variables  and , so that *β*_*ij *_=  - . The constraint Eq. 6 then has as the left side the sum over both new variables. Overall one has a linearly constrained nonlinear and nonconvex optimization problem. In principle, global optimization methods would have to be applied to find the global minimizer. Due to the dimensions of the problems these would be non-deterministic methods such as some of the many metaheuristics, which include simulated annealing and genetic algorithms. These methods require a large number of evaluations and due to their stochastic character would have to be run several times in order to increase the likelihood of finding the global optimum, although a guarantee for that is impossible. After initial tests we decided to instead use local solvers. These are very efficient and through various measures the chances of getting very good local minima can be increased substantially.

Using local solvers with "multistart" or several often randomly generated starting guesses is another way of solving global optimization problems. It is also implemented in several software packages. We instead used simple constant starting guesses. All of our results were obtained using the constant starting values of 1 or 2 for all components of β^+ ^and β^-^. We did, however, use a sequence of different t values in the regularization constraint Eq. 6 and then chose the best (local) minimum found for the solvers used. It is significant that the best solutions were obtained with values of the regularization parameter t that were small enough to restrict the fit β_ij _values. This shows that the additional L^1 ^constraint in Eq. 6 helps alleviate overfitting. In order to be able to easily call a variety of solvers we phrased the problem in the modeling language AMPL [28]. In order not to have to list many separate citations we state that we used the applicable (NLP) solvers installed at NEOS (Network Enabled Optimization Server, http://neos.mcs.anl.gov/) but run locally, not through this free service in which we (HDM) are also heavily involved.

In the way described above we generated the values in Tables 1 and 2. For Table 3 it was possible to just run the solver that had best solved the corresponding case in Table 1 on the data set and evaluate for the test sets. We did not use the optimal coefficients found but applied our method to the full training set in each case.

**Table 2:** Comparison of prediction results for the RTA method, described in this article, and the SMM-align method

	AUC		
		
MHC allele	RTA	SMM-Align	Number of data
DRB1*0101	0.748	0.702	1203
DRB1*0301	0.843	0.779	474
DRB1*0401	0.779	0.741	457
DRB1*0404	0.829	0.798	168
DRB1*0405	0.823	0.727	171
DRB1*0701	0.813	0.768	310
DRB1*0802	0.806	0.724	174
DRB1*0901	0.752	0.726	117
DRB1*1101	0.829	0.715	359
DRB1*1302	0.882	0.810	179
DRB1*1501	0.792	0.715	365
DRB3*0101	0.960	0.620	102
DRB4*0101	0.820	0.730	181
DRB5*0101	0.772	0.664	343
H2-IA^b^	0.926	0.913	76
H2-IA^d^	0.894	0.819	342
H2-IA^s^	0.891	0.877	126

The RTA results were calculated using the cross-validation sets from Ref. [18]. The SMM-align results were taken from the same reference.

**Table 3:** A comparison of peptide binding register prediction results for the peptide-MHC complexes listed in Table 5 using RTA and seven other prediction methods

		Prediction Method							
		
Class II MHC allotype	PDB entry	RTA	ARB	MHC2Pred	NN-align	ProPred	RANKPEP	SMM-align	SVRMHC
DRB1*0101	1AQD	✔	✘	✘	✔	✔	✔	✔	✔
	1DLH	✔	NA	✔	✔	✔	✔	NA	✔
	1KLG	✔	✔	✘	✔	✔	✔	✔	✘
	1KLU	✔	✔	✘	✔	✔	✔	✔	✘
	1SJE	✔	✔	✔	✔	✔	✘	✔	✔
	1T5W	✔	✔	✘	✔	✔	✔	✔	✘
	2FSE	✔	NA	✘	✔	✔	✔	NA	✘
DRB1*0301	1A6A	✔	✔	✘	✔	✔	✘	✔	NA
DRB1*0401	1J8H	✔	NA	✔	✔	✔	✔	NA	✘
	2SEB	✔	NA	✔	✘	✔	✘	NA	✘
DRB1*1501	1BX2	✔	✘	✘	✔	✔	✔	✔	✔
DRB3*0101	2Q6W	✔	NA	NA	✔	✔	NA	NA	NA
DRB5*0101	1FV1	✔	✘	✘	✔	✔	✘	✘	✔
	1H15	✔	NA	✘	✔	✔	✘	NA	✔
H2-IA^b^	1LNU	✔	NA	✘	✘	NA	✔	NA	NA
	1MUJ	✔	✔	✘	✔	NA	✔	✘	NA
H2-IA^d^	1IAO	✘	✔	✘	✘	NA	✘	✘	NA
	2IAD	✔	✔	✘	✘	NA	✘	✘	NA
**Total correct**		17/18	8/11	4/17	14/18	14/14	10/17	7/11	6/12

"✔" indicates a correct prediction and "✘" indicates an incorrect one. "NA" denotes that a prediction is not available either because the method does not have a prediction model for that MHC type or the method cannot be applied to a peptide shorter than 15 residues.

### Creating independent cross-validation sets

The most relevant measure of a prediction model's accuracy is how well it performs on novel peptide sequences that are dissimilar to those used to fit the model. A model with a large number of parameters, such as most peptide-MHC binding prediction models, can potentially overfit the data so that its accuracy is good on the training set but poor on unrelated peptide sequences. Cross-validation is one procedure that can be used to estimate the accuracy of the prediction model for novel data. We have used 5-fold cross-validation in which the data is divided into 5 approximately equal size subsets and predictions are made in turn on each subset using a model fit to the remaining data in the other 4 subsets. Because many of the peptide sequences in the experimental binding data are similar it is important to create the cross-validation sets with the fewest possible sequence similarities between subsets. The binding data from the IEDB database was divided into 5-fold cross-validation data sets using a method that minimizes the number of common 9-mer subsequences shared between different cross-validation sets while keeping the size of the 5 sets approximately the same size. The method was able to achieve perfect separation, *i.e*. no common 9-mers between sets, for all MHC allotypes except for HLA-DRB1*0101, which had only 7 common subsequences (0.12%).

We briefly outline the method here; details are given in Additional File 1. The peptide sequences are first divided into sets of similar sequences such that every sequence within a given set shares a common 9-mer with at least one other sequence in the same set but does not share any common 9-mers with sequences in other sets. If these sets are sufficiently small then perfectly separated 5-fold cross-validation sets can be created simply by partitioning these sequence sets into 5 parts, each part containing sets with approximately 1/5 of the total number of sequences. Because there are no common 9-mers between sequence sets and because each sequence set is entirely contained within a cross-validation set there are no common 9-mers between cross-validation sets. The only impediment to generating perfectly separated cross-validation sets, without any inter-set sequence similarities, is if some of the sets contain more than 1/5 of the sequences and so must be split.

The peptide sequences are divided into sets sharing common 9-mers using a graph representation of their similarities. The nodes in the graph represent sequences and edges connect nodes for sequences sharing a common 9-mer. The sequences corresponding to the nodes in each connected component of this graph are then the initial sets of similar sequences referred to above. If one of these connected subgraphs is too large then it needs to be split in two in a way that minimizes the number of similar sequence similarities between the two new parts. Since edges represent sequence similarities, this is accomplished by finding a graph cut that has few edges between the two new subgraphs. This graph cut problem is formulated as the sparsest cut problem in which the goal is to find a set of nodes S that minimizes the sparsity sp(S) defined by(9)

in which the numerator is the number of edges between nodes in S and nodes outside of S and the denominator is the minimum number of nodes in either subset. This measure insures that not only does the cut cross few edges, it also divides the graph approximately in half. It would be easy, but not very productive, to find a cut that separates only one node, for example. Furthermore we chose to solve this NP-hard problem using an approximate algorithm based on spectral graph theory. Although there are alternative algorithms that achieve tighter worst-case approximation bounds, the spectral method has worked well in applications. Also it is much easier to implement, since it only requires calculating matrix eigenvalues and eigenvectors, for which we used SVD.

The approximate solution of the sparsest cut problem is based on the bound on the sparsity(10)

in which d_max _is the maximum node degree and λ_2 _is the second smallest eigenvalue of the graph Laplacian matrix **L **defined by(11)

with d(i) the degree of node i.

This bound implies that an efficient cut, with low sparsity, is guaranteed if λ_2 _is small [29]. Furthermore, the cut can be found using the associated eigenvector **v**_2_, or so-called Fiedler vector. The cut is defined by dividing the graph into subgraphs *S *and  according to the eigenvector components with(12)

for some cutoff c [30,31]. The approximate solution to the sparsest cut problem is found by calculating the sparsity for all |V|-1 such cuts, defined by S_c _with cutoff c equal to each vector component, and choosing the one with the minimimum *sp*(*S*_*c*_).

The overall algorithm to find optimal cross-validation sets begins by grouping the peptide sequences by similarity according to connected components or, in cases in which the groups are too large, connected components split using the spectral partitioning algorithm just described. The groups only needed to be split for HLA-DRB1*0101 and only a single iteration of splitting was necessary. Each group is then iteratively added to the cross-validation set with the fewest sequences starting with the largest groups. The total number of pairs of similar sequences between the training and test sets, which ideally should be small, is equal to the total number of edges removed in the sparsest cut portion of the algorithm. This means that perfect separation is achieved for all MHC allotypes except for HLA-DRB1*0101. The generated cross-validation sets are provided in Additional File 2.

## Results and Discussion

### Binding affinity prediction

The RTA prediction method was evaluated both with the 5-fold cross-validation sets in the SMM-align reference [18], so that its performance could be compared with the results for the 9 different prediction methods examined in that paper, and also with the generally larger 5-fold cross-validation sets created using the method described above, which minimizes the overlap between training and test set peptide sequences. The prediction performance was evaluated using the area under the Receiver Operating Characteristic (ROC) curve [32], or AUC, the root mean squared error (RMSE), and the Pearson correlation coefficient. The latter two statistics were not calculated for the SMM-align data sets because the data for low affinity binders in those data sets were assigned a fixed cutoff value, which renders those statistics less meaningful. The prediction results for the new data sets are given in Table 1 and the results for the SMM-align data sets are in Table 2.

It may be seen from Table 2 that RTA had better performance than SMM-align on the same data sets for all MHC allotypes examined. The improvement is especially large for HLA-DRB3*0101, with an AUC for RTA of 0.960 compared with only 0.620 for SMM-align. However this SMM-align data set is exceptional since it contains only 3 strong binders, with IC_50 _< 500 nM, and has the highest percentage (85%) of thresholded low-affinity values (IC_50 _> 50 μM).

Ref. [18] also provides AUC values obtained by 8 other prediction methods applied to the same SMM-align data sets. The methods examined were a Gibbs sampler [12], TEPITOPE [5,6], SVRMHC [33], MHCpred [10], ARB [8], NetMHCII [34], PRED^BALB/c ^[35], and a variant of SMM-align that includes information on the peptide length and flanking residues called SMM-PRF [18]. A total of 4 of the methods could make predictions for all 14 HLA-DR allotypes, while 3 other methods could only be used for a subset of these allotypes. Only NetMHCII could be used for all 3 murine allotypes while two other methods, ARB and PRED^BALB/c^, could be applied to some of these allotypes. Overall, RTA was more accurate than the other 8 prediction methods. RTA yielded the same AUC value as TEPITOPE for HLA-DRB1*0404 and the same value as ARB for HLA-DRB1*1501. It gave a lower AUC value than one of other the other methods only for HLA-DRB1*0901, HLA-DRB1*1302, and H2-IA^s^. Significantly, RTA yielded the highest AUC values for all 9 methods examined in that study for 14 out of the 17 allotypes, even though 6 of the methods were evaluated without cross-validation so that some of the training data may have been included in the test sets.

In general, the AUC values for the IEDB data sets are slightly lower than for the corresponding SMM-align data sets for the same MHC allotypes. One factor that likely contributes to this difference is that the composition of the IEDB and SMM-align data sets are quite different. Whereas the median percentage of low affinity peptides, with IC_50 _> 50 μM, in the IEDB data set is only 1.5%, it is 52% for the SMM-align sets. One common trend between the results for the IEDB and SMM-align data sets is that the AUC for the murine MHC allotypes is generally higher than for the human ones.

The RTA prediction results for the IEDB data sets were not compared with results using other prediction methods because these other methods were fit to peptide-MHC binding data that likely significantly overlap the IEDB data, especially considering that IEDB is one of the most comprehensive databases. The occurrence of similar test set data in the training data can significantly increase the apparent prediction accuracy, leading to overly optimistic accuracy estimates. In the extreme case in which the RTA model was trained on both the training and test set data, close to perfect AUC values of > 0.995 were obtained (data not shown). This also emphasizes the importance of using minimally similar training and test data sets in order to reliably estimate the prediction accuracy for novel peptide sequences. The IEDB cross-validation sets, created using the algorithm described above, had no overlap at all, defined by identical 9-residue segments, for all but one MHC allotype. Likewise, the SMM-align cross-validation data sets used for comparison with that method had low overlap of about 0.5-2%. Finally, we point out that using more cross-validation sets, will allow a larger fraction of the limited data to be used for training but it will also generally increase the overlap between corresponding training and test sets. In the extreme case of leave-one-out cross-validation this overlap will be large, leading to overly optimistic performance estimates, unless all similar peptide sequences are removed.

The RTA method was also evaluated by making predictions for the peptide binding data in Lin *et al*. 2008 [25]. That study evaluated 21 class II MHC binding prediction servers using binding affinity data for 103 peptides binding to seven different allotypes. The peptides comprise overlapping segments of four allergens and were chosen as an unbiased test set for comparing different binding prediction methods. RTA predictions were made for all allotypes except for HLA-DRB1*0301, for which insufficient binding data was available for training. The prediction performance, as evaluated by AUC values, is shown in Table 4. A comparison with the results for the prediction servers in Ref. [25] shows that RTA yields among the most accurate results for HLA-DRB1*0101, DRB1*0301, DRB1*0701, and DRB1*1501 and about average results for HLA-DRB1*0401 and HLA-DRB1*1101. One potential uncertainty in this evaluation is how many of the peptides in this test set were used to train the individual prediction methods. As mentioned above, significant overlap between the test and training sets is expected to give an overly optimistic assessment of accuracy. This problem can be reduced through cross-validation, however this procedure is infeasible for evaluating prediction servers on the Internet.

**Table 4:** RTA prediction results for the test set of 103 overlapping antigen peptides from Lin *et al*. 2008(Ref. [25])

MHC allele	AUC
DRB1*0101	0.810
DRB1*0301	0.778
DRB1*0401	0.641
DRB1*0701	0.778
DRB1*1101	0.728
DRB1*1501	0.795

Finally, the RTA method was evaluated using the IEDB similarity reduced data sets from El-Manzalawy et al. 2008 [26]. These data sets were compiled from binding data downloaded from the IEDB database by removing similar peptides at different similarity thresholds: unique sequences (UPDS), no common 9-mer subsequences (SRDS1), and pairwise sequence identity < 80% (SRDS2). The prediction results for RTA as well as the three prediction methods evaluated in Ref. [26] are given in Tables S1-S3 in Additional File 3. These results show that the RTA method performed significantly better than the other three prediction methods. RTA had the highest AUC out of all four methods for 9/12 MHC allotypes for the UPDS data set and the highest AUC for all 12 MHC allotypes for both the SRDS1 and SRDS2 sets.

### Peptide binding register prediction

The peptide-MHC binding prediction model is based on the observation in X-ray structures of complexes that only a portion of the peptide binds to the MHC. The model predicts peptide binding at ambient temperature by summing the contributions of all possible 9-mer segments binding to the MHC. The difference between the binding affinity of the strongest binding 9-mer segment and that of other possible binding segments will be larger at the low temperatures used to determine X-ray structures and presumably is represented in the structure of the complex. The available X-ray structures of peptide-MHC complexes can be used to check this assumption by comparing the prediction of the binding register, defined by the largest term in the Boltzmann-weighted average in Eq. 7, with the actual binding register observed in the X-ray crystal structure. The peptide binding registers inferred from the X-ray structures provide additional experimental data that is unrelated to the binding affinity data and so allow an independent estimate of the model's accuracy. Finally, we note that there has been some confusion in the literature since the sequence of the peptide appearing in the X-ray crystal structure may in fact be different from the sequence of the actual co-crystallized peptide. This is because the peptide N- and C-termini may be disordered and so missing from the structure. In addition, some peptide residues with disordered side chains are modeled as alanines in the structure. Because of this potential discrepancy the peptide sequences in Table 5 were obtained from papers reporting the PDB structures rather than the structure itself.

**Table 5:** Available X-ray structures of peptide - class II MHC complexes in the Protein Data Bank.

MHC Allele	PDB Entry	Peptide Sequence
DRB1*0101	1AQD	VGSD**WRFLRGYHQ**YA
DRB1*0101	1DLH, 2G9H	PK**YVKQNTLKL**AT
DRB1*0101	1KLG	GEL**IGILNAAKV**PAD
DRB1*0101	1KLU	GEL**IGTLNAAKV**PAD
DRB1*0101	1SJE	PE**VIPMFSALS**EGATP
DRB1*0101	1T5W	AA**YSDQATPLL**LSPR
DRB1*0101	2FSE	AG**FKGEQGPKG**EPG
DRB1*0301	1A6A	LPKPPKPVSK**MRMATPLLM**QALPM
DRB1*0401	1J8H	PK**YVKQNTLKL**AT
DRB1*0401	2SEB	QY**MRADQAAGG**LR
DRB1*1501	1BX2	ENPV**VHFFKNIVT**PR
DRB3*0101	2Q6W	A**WRSDEALPL**GS
DRB5*0101	1FV1	NPVVHF**FKNIVTPRT**PPPSQ
DRB5*0101	1H15	GGV**YHFVKKHVH**ES
H2-IA^b^	1LNU	FE**AQKAKANKA**VD
H2-IA^b^	1MUJ	LPKPPKPVSK**MRMATPLLM**QALPM
H2-IA^d^	1IAO	RGI**SQAVHAAHA**EINEAGR
H2-IA^d^	2IAD	RGHN**TNGVTAASS**HE

The 9-residue core segment of the peptide that binds to the MHC molecule, which begins with the P1 residue, is highlighted in boldface in the peptide sequence. These structures show which peptide segment binds to the MHC molecule and so are used to evaluate how well the RTA method predicts the correct peptide binding register.

Peptide binding register predictions were made for all unique peptide-MHC complexes with available X-ray structures. These are shown in Table 5. The 9-mer segment in the structure begins with the residue binding in the P1 pocket and the predicted 9-mer segment extends from residue *M*_max _+1 to *M*_max _+ 9 in which *M*_max _is the *M *index of the largest term in the Boltzmann sum appearing in Eq. 7. All peptides similar to those for which the predictions were made were removed from the training set. The results showed that the RTA method predicted the peptide binding registers accurately. The predictions for all peptides in Table 5 were correct except for one, the peptide RGISQAVHAAHAEINEAGR binding to H2-IA^d ^from PDB entry 1IAO. Interestingly, the core segment for the CLIP peptide is the same for binding to both HLA-DRB1*0301 (PDB entry 1A6A) and H2-IA^b ^(PDB entry 1MUJ), as has been observed for most other human and murine MHC allotypes [36,37]. CLIP binds to all class II MHC molecules as an intermediate step in MHC processing and peptide loading.

For comparison, peptide binding register predictions were made for the peptide-MHC complexes listed in Table 5 using seven other prediction methods implemented as web servers: ARB [8], MHC2Pred [38], NN-align [39], ProPred [6], RANKPEP [9], SMM-align [18], and SVRMHC [33]. Each of these methods uses different computational approaches to predicting peptide-MHC binding affinities. ARB, ProPred, and RANKPEP use position specific scoring matrices (PSSMs), SVRMHC and MHC2Pred use Support Vector Machines (SVMs), NN-align uses artificial neural networks (ANNs), and SMM-align uses a combination of scoring matrices and a novel Gibbs sampler method [12]. The overall prediction results for RTA and these other methods are given in Table 3. The performance of the different prediction methods could not be directly compared since most methods could not be applied to all peptide-MHC complexes. However, RTA is clearly among the most accurate methods for this data set, with 17 out of the 18 peptide binding cores correctly predicted. A recent study by Wang *et al*. [40] performed a similar analysis using an overlapping but slightly different set of peptide-MHC complexes and obtained qualitatively similar results for the same prediction methods examined in that study. That study concluded that the TEPITOPE method [5], as implemented by the ProPred web server [6], achieved the best overall performance. We found similar results, with only RTA and ProPred correctly predicting all 14 HLA-DR peptide binding registers. NN-align achieved similar performance with 13 correctly predicted HLA-DR peptide binding cores. Significantly, some prediction methods, which generally attain accuracies that are comparable to or better than the earlier TEPITOPE method in predicting peptide binding affinities, had significantly worse accuracy than TEPITOPE in predicting the peptide binding cores. This demonstrates that a prediction model that achieves high accuracy for peptide binding affinity does not necessarily achieve corresponding high accuracy for predicting the peptide binding core. The fact that our RTA method does perform well for both prediction tasks suggests that the underlying approximate physical model for the contribution of each binding register to the total binding affinity is correct and accurate.

### Importance of suboptimal terms in the Boltzmann sum

A common approximation in other prediction models, such as TEPITOPE, is to use only the peptide binding register with the highest affinity and so neglect the contributions of suboptimal binding registers. Under this approximation Eq. 7 would become(13)

**Figure 1:**
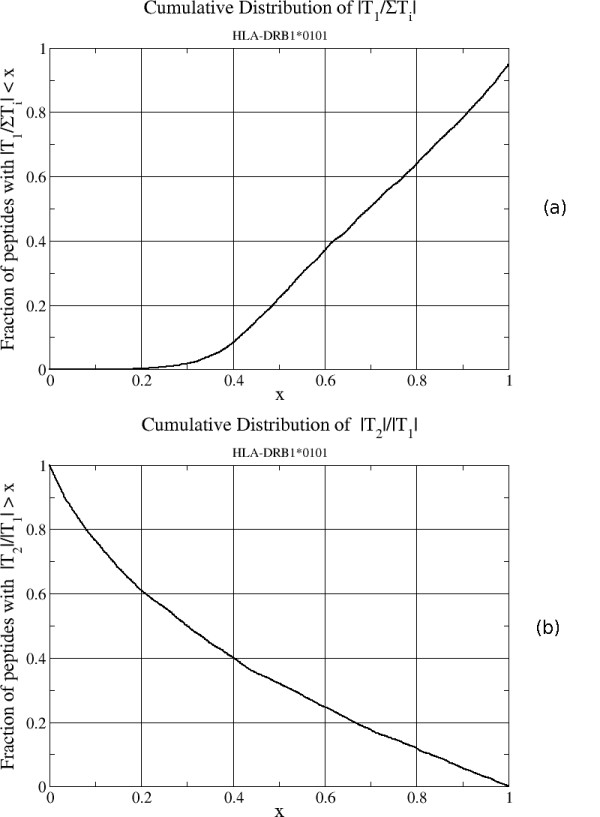
**the cumulative distribution of the magnitudes of terms in the Boltzmann sum**. (a) shows the cumulative distribution of the magnitudes of the ratio of the largest term in the Boltzmann sum in Eq. 7 to the total sum, . (b) shows the cumulative distribution of the magnitudes of the second largest term to the largest term in the Boltzmann sum, |*T*_2_/*T*_1_|. The distributions were calculated using the 5648 peptides in the IEDB data set for HLA-DRB1*0101.

This approximation is accurate if only one term dominates the Boltzmann average in Eq. 7. In order to estimate the importance of the suboptimal terms included in our model, we calculated the ratios  and |*T*_2_/*T*_1_|, in which *T*_*i *_are the terms in the Boltzmann sum in Eq. 7 ordered so that |*T*_1_| ≥ |*T*_2_| ≥ L ≥ |*T*_*L*-8_|. *T*_1 _(*T*_2_) are then the terms with the largest (2^nd ^largest) absolute values. Both ratios vary from 0.0 to 1.0. These ratios were calculated for the largest IEDB data set, containing 5648 peptides binding to HLA-DRB1*0101. A low value of  indicates that suboptimal terms make a significant contribution to the sum so that the approximation using only the optimal term is inaccurate. The cumulative distribution of this quantity is shown in Figure 1(a). For example, suboptimal terms contribute at least 50% to the Boltzmann sum for about 20% of the peptide sequences. Likewise Figure 1(b) shows the cumulative distribution for the relative contribution of the leading suboptimal term relative to the optimal term, |*T*_2_/*T*_1_|. For example, it can be seen that this ratio is greater than 0.4 for about 40% of the sequences. The ratios for other data sets are expected to have a similar distribution. To the extent that these predicted values are accurate, the distributions of these ratios indicate that suboptimal terms make significant contributions to the total binding affinity for many peptides so that the assumption that only one binding register dominates is incorrect for these peptides. The error in the predicted binding affinity for these peptides in a prediction model including only the optimal term may not be as large as expected since the model parameters are fit directly to experimental binding affinities and so the model may compensate to some degree for the contribution of missing suboptimal terms. In any case, to the extent that these estimates of the contributions of suboptimal terms are correct, prediction models, such as RTA, that incorporate these terms are expected to be more accurate.

## Conclusions

The prediction results for the RTA method show (1) that suboptimal peptide binding registers make a significant contribution to the total peptide binding affinity for many peptides, (2) regularization with the L^1 ^parameter constraint improves prediction accuracy for novel data, (3) the method achieves good accuracy in predicting peptide-MHC binding affinities with generally higher AUC values than other prediction methods evaluated on the same data sets, and (4) the method accurately predicts the 9-residue binding core for all but one out of 18 peptide-MHC complexes examined. While peptide-MHC binding affinities can be quickly calculated using Eq. 7, the efficient solution of the difficult global optimization problem for fitting parameters using local optimization methods contributed to the success of the method. The accuracy of the method is expected to continually improve as more experimental binding data become available, particularly for certain MHC allotypes with limited current data.

One direction for future work is to extend the RTA model to a multi-allotype model by including another parameter dimension reflecting the MHC peptide binding determinants, either through unique binding pockets, as in TEPITOPE, or through polymorphic pocket residues, as in NetMHCIIpan [41]. Another possible extension is to include interaction terms for nearby peptide residues. The approximation that contributions of individual peptide residues to the total binding free energy are largely independent is supported by side chain scanning experiments [3] and by the accuracy of prediction models, such as RTA, that are based on this approximation. However, recent experimental studies [42,43] have found evidence for cooperative effects between peptide residues so that additional pairwise interaction terms may improve prediction accuracy. Both of these modifications will significantly increase the number of model parameters, making regularization even more crucial for prediction accuracy.

## Authors' contributions

AJB conceived of the study, collected the data sets, and analyzed the prediction results. HDM performed the numerical optimization to determine the model parameters. Both authors participated in drafting the manuscript and read and approved the final manuscript.

## Supplementary Material

Additional file 1 Detailed description of the algorithm for creating cross-validation data sets with minimal peptide sequence overlap. Click here for file

Additional file 2 This zip archive contains tab-separated files with the 5-fold cross-validation sets for 16 different MHC allotypes created for this study and also a separate text file describing the table format. Click here for file

Additional file 3 Tables S1-S3 present the prediction results for the IEDB benchmark sets from El-Manzawaly *et al*. 2008 [26]. Prediction accuracies are given for RTA as well as the three prediction methods studied in that paper. Click here for file
