# Supplementary material for: Prediction of the binding affinities of peptides to class II MHC using a regularized thermodynamic model
Source: BMC Bioinformatics. 2010 Jan 20;11:41. doi: 10.1186/1471-2105-11-41 (PMC2828437; doi:10.1186/1471-2105-11-41)
Supplement: Additional file 1 — Detailed description of the algorithm for creating cross-validation data sets with minimal peptide sequence overlap. [file 1471-2105-11-41-S1.PDF]

Description of the algorithm for creating cross-validation sets with minimal overlap

The following is pseudocode for the method used to create k-fold cross-validation test sets with minimal overlap:

Create a graph G with peptide sequences represented by nodes and with edges between all pairs of nodes for sequences with common 9-residue subsequences

Find the connected components of the graph $\left\{C_i, i = 1, \ldots, N_{\text{components}}\right\}$

Sort the connected components in descending order by size so that $\left|C_i\right| \geq \left|C_j\right|$ for $i < j$

Initialize set of graphs G to be the connected components, *i.e.* $G = \left\{C_i, i = 1, \ldots, N_{\text{components}}\right\}$

Calculate the maximum allowable sequence group size $N_{\max} = \lfloor N_{total} / k \rfloor$, where there are a total of N_{total} unique peptide sequences

while ($\max\left(\left\{\left|G_i\right| : G_i \in G\right\}\right) > N_{\max}$) {

Set $G_{\max} = \arg\max\left(\left\{\left|G_i\right| : G_i \in G\right\}\right)$, *i.e*. the largest graph in G

Calculate the minimum node subset size $N_{\min} = \left|G_{\max}\right| - N_{\max}\left(\left\lceil\left|G_{\max}/N_{\max}\right|\right\rceil - 1\right)$

// Find the sparsest cut of $G_{\max}$ using spectral graph theory
Calculate the Laplacian $\mathbf{L}$ of $G_{\max}$

Calculate the eigenvalues , λ_j , and associated eigenvectors, $\mathbf{v}_j$, of $\mathbf{L}$, and sort them so that $\lambda_1 \leq \lambda_2 \leq \cdots \leq \lambda_N$

Order the nodes in $G_{\max}$ according to the components of $\mathbf{v}_2$ so that every pair of nodes n_i and n_j with $i < j$ has $\left(\mathbf{v}_2\right)_i \geq \left(\mathbf{v}_2\right)_j$ for the corresponding eigenvector components

Initialize BestRatio to an arbitrary large value

for (k = 1 to $\left|G_{\max}\right|$) {

Define graphs G_{sub1} and G_{sub2} as the subgraph of $G_{\max}$ containing the nodes $\left\{n_i, i \leq k\right\}$ and $\left\{n_i, i > k\right\}$, respectively

Calculate $\text{Ratio} = \frac{\text{Number of edges connecting } G_{sub1} \text{ and } G_{sub2}}{\min\left(\left|G_{sub1}\right|, \left|G_{sub2}\right|\right)}$

if (Ratio < BestRatio) {

BestRatio = Ratio

$T_1 = G_{sub1}$, $T_2 = G_{sub2}$

}

}

Cut $G_{\max}$ by replacing it in G with T_1 and T_2

}

Define S_i to be the sequences associated with the nodes for each $G_i \in G$, $i = 1,\ldots,|G|$ and order them by decreasing size so that $|S_1| \geq |S_2| \geq \cdots > |S|_{|G|}$

// Sequentially add largest sequence group to currently smallest cross-validation set

Initialize cross-validation sets $CV_i = \varnothing$ for $i = 1,\ldots,k$

for (i = 1 to $|G|$) {

Define $j = \arg\min\left(\left\{|CV|_i, i = 1,\ldots,k\right\}\right)$, *i.e.* index of the currently smallest cross-validation set

Add sequences S_i to CV_j, *i.e.* $CV_j = CV_j \cup S_i$

}
