# Supplementary material for: Prediction of the binding affinities of peptides to class II MHC using a regularized thermodynamic model
Source: BMC Bioinformatics. 2010 Jan 20;11:41. doi: 10.1186/1471-2105-11-41 (PMC2828437; doi:10.1186/1471-2105-11-41)
Supplement: Additional file 3 — Tables S1-S3 present the prediction results for the IEDB benchmark sets from El-Manzawaly et al. 2008 [26]. Prediction accuracies are given for RTA as well as the three prediction methods studied in that paper. [file 1471-2105-11-41-S3.PDF]

Table S1. Prediction results for RTA and three other methods for the IEDB-UPDS benchmark data sets of El-Manzalawy et al. 2008. The highest AUC value for each MHC allotype is highlighted in bold.

	**AUC**			
**MHC allotype**	**RTA**	**5-spectrum**	**LA**	**CTD**
DRB1*0101	0.753	**0.806**	0.767	0.770
DRB1*0301	**0.828**	0.771	0.822	0.788
DRB1*0401	**0.797**	0.769	0.786	0.790
DRB1*0404	**0.907**	0.639	0.763	0.821
DRB1*0405	**0.895**	0.693	0.770	0.788
DRB1*0701	**0.847**	0.727	0.799	0.757
DRB1*0802	**0.907**	0.703	0.896	0.793
DRB1*1101	0.815	0.800	**0.822**	0.798
DRB1*1302	**0.895**	0.783	0.865	0.775
DRB1*1501	**0.861**	0.794	0.781	0.779
DRB4*0101	**0.857**	0.703	0.795	0.738
DRB5*0101	0.777	0.784	0.755	**0.785**

Table S2. Prediction results for RTA and three other methods for the IEDB-SRDS1 benchmark data sets of El-Manzalawy et al. 2008. The highest AUC value for each MHC allotype is highlighted in bold.

	**AUC**			
**MHC allotype**	**RTA**	**5-spectrum**	**LA**	**CTD**
DRB1*0101	**0.724**	0.427	0.628	0.604
DRB1*0301	**0.806**	0.460	0.668	0.605
DRB1*0401	**0.765**	0.444	0.619	0.671
DRB1*0404	**0.899**	0.411	0.644	0.703
DRB1*0405	**0.889**	0.331	0.557	0.654
DRB1*0701	**0.818**	0.445	0.658	0.605
DRB1*0802	**0.883**	0.378	0.762	0.692
DRB1*1101	**0.790**	0.454	0.668	0.698
DRB1*1302	**0.850**	0.549	0.750	0.627
DRB1*1501	**0.836**	0.460	0.595	0.623
DRB4*0101	**0.834**	0.444	0.611	0.547
DRB5*0101	**0.750**	0.344	0.578	0.638

Table S3. Prediction results for RTA and three other methods for the IEDB-SRDS2 benchmark data sets of El-Manzalawy et al. 2008. The highest AUC value for each MHC allotype is highlighted in bold.

	**AUC**			
**MHC allotype**	**RTA**	**5-spectrum**	**LA**	**CTD**
DRB1*0101	**0.718**	0.436	0.629	0.664
DRB1*0301	**0.795**	0.401	0.657	0.673
DRB1*0401	**0.757**	0.371	0.592	0.662
DRB1*0404	**0.892**	0.364	0.578	0.664
DRB1*0405	**0.889**	0.340	0.523	0.576
DRB1*0701	**0.819**	0.389	0.611	0.609
DRB1*0802	**0.882**	0.370	0.732	0.732
DRB1*1101	**0.795**	0.399	0.602	0.666
DRB1*1302	**0.858**	0.483	0.681	0.594
DRB1*1501	**0.812**	0.380	0.534	0.636
DRB4*0101	**0.834**	0.466	0.627	0.644
DRB5*0101	**0.752**	0.278	0.503	0.492
